# Hydroxy decenoic acid down regulates *gtfB* and *gtfC* expression and prevents *Streptococcus mutans* adherence to the cell surfaces

**DOI:** 10.1186/1476-0711-11-21

**Published:** 2012-07-28

**Authors:** Behnam Yousefi, Shahrooz Ghaderi, Alireza Rezapoor-Lactooyi, Niusha Amiri, Javad Verdi, Alireza Shoae-Hassani

**Affiliations:** 1 School of Advanced Medical Technologies, Tehran University of Medical Sciences (TUMS), Tehran, Iran; 2 School of Medicine, Tabriz University of Medical Sciences, Tabriz, Iran; 3 Periodontology Department, Shahid Beheshti University of Medical Sciences, Tehran, Iran; 4 Department of Applied Cell Sciences, School of Advanced Technologies in Medicine, Tehran University of Medical Sciences (TUMS), Tehran, Iran; 5 Tissue Engineering and Stem cell department, Research center for Science and Technology in Medicine (RCSTiM), Tehran University of Medical Sciences (TUMS), P.O.Box: 1998896953, Tehran, Iran

**Keywords:** Biofilm, Caries, Glucosyltransferase, Streptococcus

## Abstract

**Background:**

10**-**Hydroxy-2-decenoic acid, an unsaturated fatty acid is the most active and unique component to the royal jelly that has antimicrobial properties. *Streptococcus mutans* is associated with pathogenesis of oral cavity, gingivoperiodontal diseases and bacteremia following dental manipulations. In the oral cavity, *S. mutans* colonize the soft tissues including tongue, palate, and buccal mucosa. When considering the role of supragingival dental plaque in caries, the proportion of acid producing bacteria (particularly *S. mutans*), has direct relevance to the pathogenicity of the plaque. The genes that encode glucosyltransferases (*gtf*s) especially *gtfB* and *gtfC* are important in *S. mutans* colonization and pathogenesis. This study investigated the hydroxy-decenoic acid (HDA) effects on *gtfB* and *gtfC* expression and *S. mutans* adherence to cells surfaces.

**Methods:**

*Streptococcus mutans* was treated by different concentrations of HPLC purified HDA supplied by Iran Beekeeping and Veterinary Association. Real time RT-PCR and western blot assays were conducted to evaluate *gtfB* and *gtfC* genes transcription and translation before and after HDA treatment. The bacterial attachment to the cell surfaces was evaluated microscopically.

**Results:**

500 μg ml^-1^ of HDA inhibited *gtfB* and *gtfC* mRNA transcription and its expression. The same concentration of HDA decreased 60% the adherence of *S. mutans* to the surface of P19 cells.

**Conclusion:**

Hydroxy-decenoic acid prevents *gtfB* and *gtfC* expression efficiently in the bactericide sub-concentrations and it could effectively reduce *S. mutans* adherence to the cell surfaces. In the future, therapeutic approaches to affecting *S. mutans* could be selective and it’s not necessary to put down the oral flora completely.

## Background

Oral streptococci are important components of the complex oral biofilm known as dental plaque. Members of the *Streptococcus* genus including *Streptococcus mutans* are associated with dental caries [1,2]. In the oral cavity, organisms colonize the tongue, palate, and buccal mucosa [3,4]. *Streptococcus mutans* strains have been recovered from the subgingival crevice, a well studied microbial niche [5-7]. The ability of bacteria to colonize the different oral surfaces depends on their binding potential. When considering the role of supragingival dental plaque in dental caries, the proportion of gram positive acid producing bacteria (particularly *S. mutans*), has direct relevance to the pathogenicity of the plaque. These microorganisms tolerate a low pH environment, and thrive in cariogenic substrates such as sucrose [8]. The most frequent oral infections include gingivoperiodontal diseases including gingivitis and periodontitis, are caused by dental plaque, which is a *S. mutans* produced biofilm [9-11]. The primary mechanism for adherence of *S. mutans* is the production of glucan polymers from sucrose via glucosyltransferases (Gtf) [12] that is an essential virulence factor associated with the pathogenesis of *S. mutans*[13]. Hence, the factors influencing expression of *gtf* genes are very important for prevention of dental plaques, caries, gingivitis, gingival abscess and even bacteremia following dental manipulation. Glucosyltransferases encoded by *gtfB* and *gtfC* genes show similarities. GtfB is an exoenzyme involved with the extracellular metabolism of Sucrose [14]. GtfB synthesizes a polymer of mostly insoluble (α-1,3-linked) glucan and GtfC synthesizes a mixture of insoluble (α-1,3-linked) and soluble (α-1,6-linked) glucans [15,16]. These glucans are important components of the matrix of cariogenic biofilms.

The pH of the all experiments set between (7–7.5). *Significant differences were tested by analysis of variance (ANOVA). p ≤0.05.

**Figure 1:**
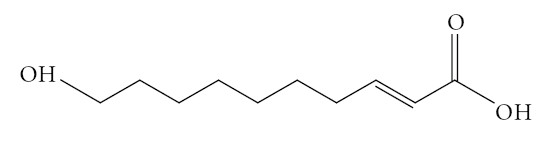
The structure of Trans 10-Hydroxy-2-Decenoic acid (HDA) from Royal jelly.

10-Hydroxy-2-decenoic acid (HDA) is an important part of royal jelly. Royal jelly (RJ) so called because it is the exclusive food of the Queen bees, which secreted by the hypopharyngeal and mandibular glands of *Apis mellifera* bees to feed the queen [17]. RJ is a natural source of essential amino acids, lipids, vitamins, acetylcholine, and many other nutrients [18,19]. It has a wide range of medical activities such as antimicrobial effects [20,21] and preventing cell damage in cancer and HIV patients [22,23]. The potency of antibacterial properties of RJ is related to HDA [24,25], a bioactive component that occupies 10% of the RJs total weight. The structure of HDA is depicted in Figure 1. HDA is capable to induce the dispersion of *S. mutans* biofilm microcolonies. HDA is highly acidic and acts as a detergent and antimicrobial agent [26]. It has antitumor [27] and collagen production activities [28]. It is known as a safe natural product, thus here we investigated the HDA effect on *gtfB* and *gtfC* expression and consequently adherence of *S. mutans* colonies on the eukaryotic cell surfaces. 

## Methods

### Preparation of HDA and bacterial treatment

HPLC purified 10-hydroxy-2-decenoic acid (Figure 1) were provided by Iran Beekeeping and Veterinary Association (Tehran, Iran). *Streptococcus mutans* ATCC 25175 was purchased from Persian Type Culture Collection (PTCC, Tehran, Iran). The strain was cultured in Brain Heart Infusion broth, (BHIB Difco, Detroit, USA) at 37°C with 5% defibrinated sheep blood in an atmosphere containing 5% CO_2_. For treatment the cultures were supplied by addition of 100 mM sucrose (Merck, Germany). The early exponential phase cultures (pH 6.8) were treated with 100, 200, 500 and 1000 μg ml^-1^ of ethanol dissolved HDA (treatment in peptone buffer, Difco) in 2 ml microtubes during 8 hours and then transferred to the previous media. Untreated peptone water was used as a control. The bacterial growth was determined by measuring the optical densities (OD) at 600 nm. OD was monitored at 1 h intervals. From the point that HDA is an acidic compound and it may kill the bacteria by the pH changes we controlled the pH (7–7.5) by NaHCO3 and NaOH buffer. The treated cells extracted by centrifugation (10 min at 1000 *g*) for other examinations. The culture supernatant was also examined for GtfB/GtfC extracellular enzyme analysis.

### Time-kill assays

Time-kill study was performed by the broth dilution method [29]. The inoculum of *S. mutans* was 1^×^10^6^ CFU/ml. The final concentration of the HDA was four times the MIC (2000 μg ml^-1^). Tubes containing the microorganisms and the HDA in BHIB were incubated in 5% CO_2_ at 37°C; samples were removed for determination of viable counts at 30 min and 1, 2, 4, 8, and 24 h. Serial dilutions (10^-1^ to 10^-4^) were prepared in sterile saline solution. The diluted sample (50 μl) was plated onto Brain heart infusion agar (BHIA) with a spiral plater (Model 3000; Spiral Biotech, Bethesda, US). The plates were incubated in 5% CO_2_ for 48 h, when the number of colonies was determined. Killing curves were constructed by plotting the log10 CFU per milliliter over 24 h. All of the assays were done in triplicate.

### Evaluation of *gtfB* and *gtfC* expression via Real time RT-PCR

Total RNA from treated cultures of *S. mutans* were extracted and purified using the RNeasy kit (Qiagen, Germany) followed by digestion with RNase free DNase-I according to the manufacturer’s instruction. The cDNA were synthesized using a cDNA synthesis kit (Bio-Rad Lab, US). To check for DNA contamination, purified RNA without reverse transcriptase served as a negative control. The expression of related genes was quantified using the SYBR green reagent (2X SYBR Green Supermix; Bio-Rad, CA) following the instructions of the manufacturer on a Bio-Rad iCycler. PCR was performed in multiplicate in optimized conditions: 95°C denatured for 3 min, followed by 40 cycles of 45 s at 94°C, 45 s at 55°C, and 45 s at 72°C using the following primers: *gtfB* (F: 5´ -CGAACAGCTTCTAATGGTGAAAAGCTT- 3´, R: 5´-TTGGCTGCATTGCTATCATCA-3´) and *gtfC* (F: 5´-GCCACGGAACAAGCAGTTCTGTAA- 3´, R: 5´-TAATACCAATTATTTCCTAAGCTAA-3´)(NCBI sequence Ref No. NC-004350). Fluorescence signals were measured over 40 PCR cycles. The cycle number (*Ct*) at which the signals crossed a threshold set within the logarithmic phase was recorded. For quantitation, we evaluated the difference in cycle threshold (Δ*Ct*) between the treated group and vehicle control of each gene. The efficiency of amplification of each pair of primers was determined by serial dilutions of templates and all were >0.9. Each sample was normalized with the loading reference 16 S rRNA (NCBI sequence Ref No. X58303). Experiments were repeated at least three times.

### Western blot analysis of GtfB and GtfC

*Streptococcus mutans* cultures grown with the various concentrations of HDA (100–1000 μg ml^-1^) were centrifuged 5 min at 5000 *g*. The pellets were resuspended in Tris HCl 30 mM, pH 8.1 and centrifuged 10 min at 10000 *g*. The pellets were vortexed in 200 μl sucrose 20% in Tris HCl. These cells were resuspended in phosphate buffer (pH 7) and were incubated on ice with 33 mg/L lysozyme for 30 min and then were disrupted by sonication for 20s. After centrifugation for 15 min at 15000 *g*, 100 μl aliquot of the supernatant was mixed in sample buffer as described previously [30] on 15% polyacrylamide gel electrophoresis. Final detection of GtfB and GtfC enzymes was driven by western blot analysis using goat anti-rabbit IgG conjugated with HRP (Dakopatts, Glostrup, Denmark).

### *Streptococcus mutans* adherence assay to P19 cells and antibiotic protection assay

P19 embryonic cells purchased from Pasture Institute cellular bank (Tehren, Iran) were maintained in Dulbecco’s modified Eagle’s Medium (DMEM,Gibco, UK) supplemented with 10% fetal calf serum (FCS, Gibco, UK). Cells were plated at 10^5^ cells/well in six well plates coated with collagen. After 1 day of incubation, the cells were examined under a phase-contrast microscope for morphological changes. Five areas, each containing minimum 100 cells, were randomly selected in each well, and were counted. Cell proliferation was evaluated by counting the total cell number after treatment with different concentrations of HDA [31]. Prior to infection, *S. mutans* (10^6^ CFU per ml in BHI) were mixed either with 100 ml FCS and 100 ml BHI and incubated at 37°C for 1 h. The bacterial mixtures were centrifuged at 5000 *g* for 5 min. The pellets were washed once with PBS and resuspended in DMEM. To determine the number of bacteria that were able to reach inside the cell, antibiotic protection assay was performed as described previously [32]. After 2 h of co-culturing P19 with *S. mutans*, the wells were washed three times with fresh DMEM/F12 without antibiotics to remove planktonic bacteria. One milliliter of DMEM/F12 containing 300 *μ*g/ml gentamicin and 10 *μ*g/ml penicillin was added to the wells and incubated for 3 h at 37°C in a 5% CO2 atmosphere to eliminate extracellular bacteria. Next, the wells were washed 3 times with PBS without antibiotics and the P19 cells were lysed by PMSF in 1 ml of dH_2_O for 20 min. The mixture of lysed P19 cells and free bacteria was collected from the wells and serially diluted in PBS, followed by plating onto BHI agar and incubation at 37°C in a 5% CO2 atmosphere. After 2 days, the CFUs were counted and the percentage of intracellular bacteria relative to the initial inoculum was calculated. Each experiment was performed in triplicate under standard conditions.

### Statistical analysis

The analysis of data was performed by ANOVA using SPSS 11.0. Differences were considered to be statistically significant when a value of P≤0.05 was obtained.

## Results

### Time kill kinetic assay

**Figure 2:**
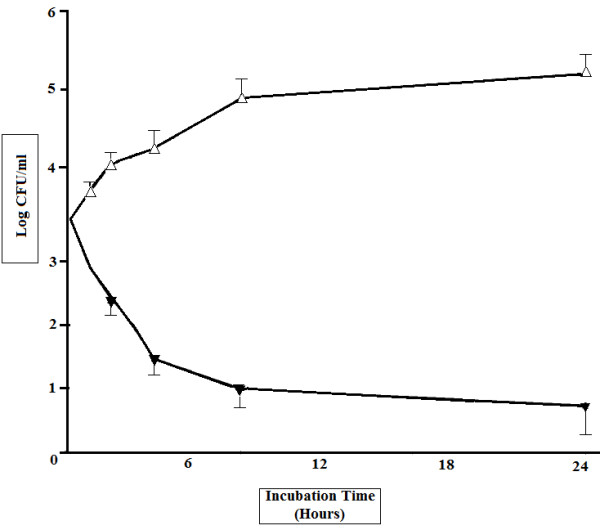
**Time-kill curve for *S. mutans *strain by hydroxy-decenoic acid at 4 times of the MIC (data not shown).** Symbols: ▴: Test group and Δ: control group of *S. mutans* ATCC 25175.

The result of the time-kill study is shown in Figure 2. HDA in 1000 μg ml^-1^ rapidly reduced the viable counts of *S. mutans* within 1 h of incubation (reduction of 1 log in the number of CFU). It has shown bactericidal effects (a >3log decrease in the CFU) on *S. mutans* for 8 h of incubation (Figure 2). All the experiments were in pH ranged between 7–7.5 by the buffer system as described in methods.

### Real Time RT PCR analysis

**Figure 3:**
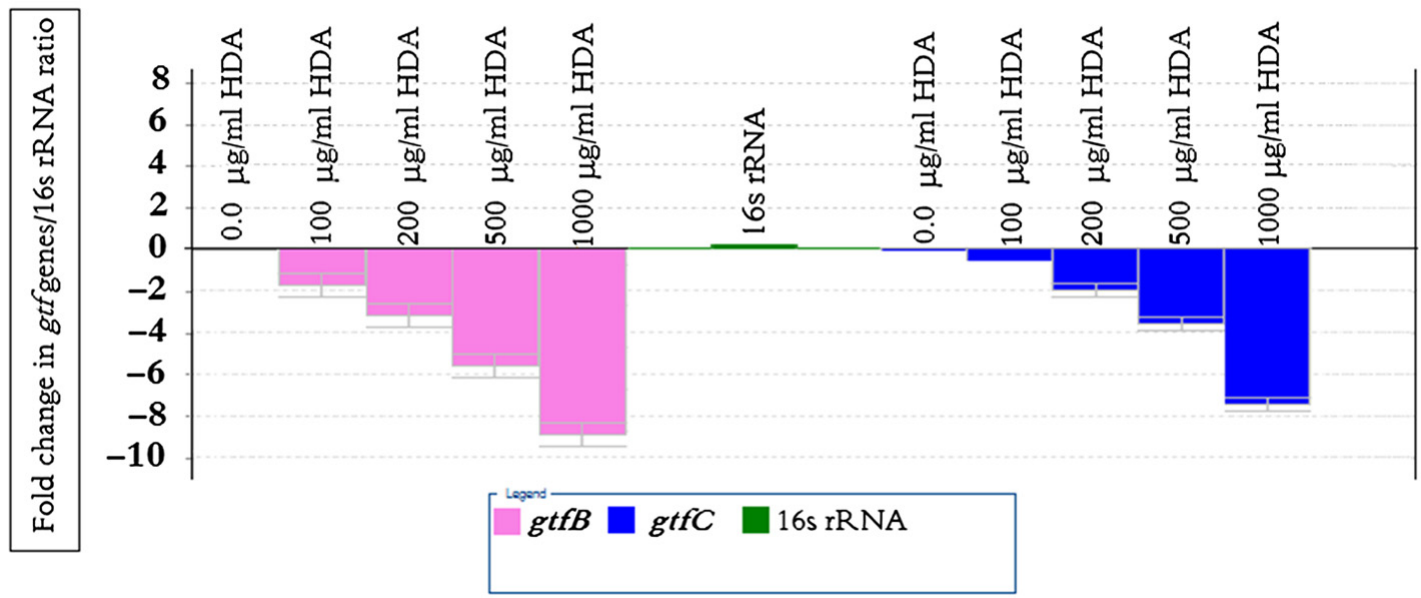
**Transcription of *gtfB/gtfC *from *S. mutans *following HDA treatment. ***gtfB/gtfC* expression is completely abrogated by exposure to HDA. *gtfB/gtfC* levels are also reduced with lower concentrations of HDA. The error bars represent mean and standard deviations of experiments performed in triplicate. *gtf* genes were more abundantly expressed in cultures that treated with 0.0, 100 and 200 μg ml^-1^ of HDA but missing in the cells treated with 1000 μg ml^-1^ of HDA.

We performed real time RT-PCR experiments to examine the abundance of *gtfB* and *gtfC* specific mRNA in *S. mutans* cells treated with HDA (Figure 3) Equal amounts of total RNA from early exponential phase cultures were used to reveal the transcript levels of *gtfB/gtfC* before and after HDA treatments. The analysis revealed that the *gtfs* were more abundantly expressed in untreated cultures. No significant difference of *gtfC* mRNA transcripts was observed among different treatment groups from 0.0 to 100 μg ml-1 of HDA. While 500 μg ml-1 of HDA greatly inhibited *gtfB and gtfC* transcription (Figure 3).

### Glucosyltransferases analysis by Western blot

**Figure 4:**
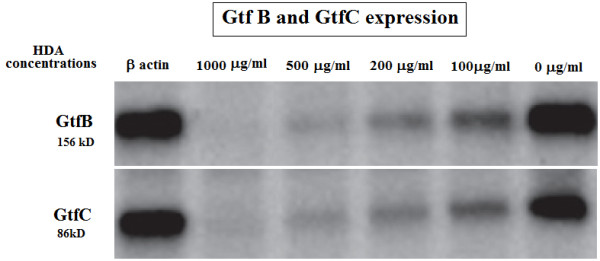
**Western blot analysis of GtfB and GtfC enzymes.** GtfB and GtfC proteins that are present in *S. mutans* treated with 0.0, 100, and 200 μg ml^-1^ concentrations of HDA but are missing in the cells treated with 500 and 1000 μg ml^-1^ or higher concentration of HDA. The 16SrRNA protein was used as control.

In order to verify whether change in expression of the *gtfB/gtfC* at the transcriptional level could be duplicated at the protein levels, the intra and extracellular proteins were prepared from cultures of bacteria grown in the enriched BHI medium before and after HDA treatment analyzed by the western blot using goat anti-rabbit IgG. The strength of GtfB and GtfC bands of different treatment groups were revealed as shown in Figure 4. Concentration of 500 μg ml^-1^ of HDA could repress the production of Gtfs completely but the Gtfs production was observed in the samples treated with 200 μg ml^-1^ or lower concentrations as expected from transcription analysis (Figure 4). Therefore, the expression of *gtfB* and *gtfC* in response to HDA was consistent at the transcriptional and translational levels.

### Adherence to P19 cells

**Figure 5:**
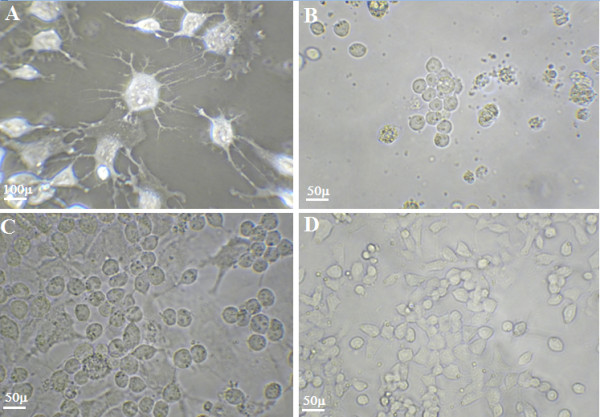
**Cultures f P19 embryonal carcinoma monolayer cells: after treatment with HDA disrupted the organization of the monolayer of P19 cells (A); and addition of *S. mutans *to monolayer of P19 cells that disrupted the organization of monolayer to a greater extension (B); *S. mutans *treated with HDA in P19 cultures (C) and not treated culture (D).** Magnification in part A is 600X and in the all other images is X400.

It was obvious that HDA could slightly reduce P19 embryonal carcinoma cell proliferation and there is a significant difference when compared with PBS control cultures (Figure 5A,B). Also HDA could differentiate the cells phenotype into neural cells but it didn’t show major cytotoxicity effects on the cells (Figure 5A) as compared by intact P19 cultures (Figure 5D). Our examination showed that 500 μg ml^-1^ of HDA prevented adhesion of *S. mutans* to the P19 cell surfaces effectively (Figure 5C) as compared by untreated *S. mutans* cultures (Figure 5B). Concentrations of 100, 200, 500 and 1000 μg ml^-1^ of HDA prevent 12, 31, 59 and 61% of *S. mutans* cells from adherence to P19 embryonic cells, respectively (Table 1) that determined by gram staining of six well plate cultures.

**Table 1:** **Inhibitory effect of HDA on *S. mutans *adherence to the P19 cell**

**HDA concentration**	**Log of Cell count**	**Percent of preventive**
**μg ml^-1^**	**per Well**	**adherence**
0.00	6	0.00
100	5.28	12
200	4.14	31
500	2.46	59
1000	2.34	61

The pH of the all experiments set between (7–7.5). *Significant differences were tested by analysis of variance (ANOVA). p≤0.05.

## Discussion

There are evidences that GtfB and GtfC enzymes are the most important Gtfs related to dental caries [33]. The large size of the *gtf* genes made transcriptional analysis troublesome, but we decided to investigate *gtfB* and *gtfC* genes expression after treatment of the bacterial cultures with a natural antimicrobial compound called HDA. In the present study, it was found that HDA inhibited *gtfB* and *gtfC* mRNA transcription and expression. Also it was a good adherence inhibitor of *S. mutans*.

It was found that the nutrient content of the media culture regulates the progression of biofilms in organisms [34]. Biosynthesis of glucan polymers is critical for the adherence of *S. mutans* to the surfaces; hence, we tested HDA effect on *S. mutans* attachment quality to the eukaryotic cell surfaces. Because the levels of mRNA is significantly higher in early than in the late exponential phase [35], our study was done based on early exponential phase of *S. mutans* cultures. Despite the fact that our model does not resemble exactly the microbial community found in dental plaques, yet it is profitable model for our investigations.

It was established that the glycolysis and fermentation yield acids that can acidify the biofilms and increase the availability of sucrose [36]. Differential analysis of the S. mutans grown in various nutrient revealed alterations in the genes expression involved in biofilm formation. Also production of glycolytic enzymes could regulate the expression of *gtf* genes [37]. The *gtf* genes are induced in response to decreasing pH of the biofilms and/or in response to the presence of a metabolizable sucrose [38]. Studies on *S. mutans*, using real time RT-PCR, showed two fold increase of *gtf* mRNA expression in the presence of sucrose [39]. Our real time RT-PCR assay revealed significant decrease in *gtfB* and *gtfC* expression after HDA treatment in spite of sucrose addition (Figure 3), supporting HDA as a negative transcriptional regulator of the sucrose dependent activity. However the concentration of sucrose has influence in the pH and *gtf* genes regulation [40] the present results showed that the pH effect can be reversed by the HDA treatment (Figure 3).

The influence of HDA on *gtf * transcriptional levels also affected the levels of GtfB and GtfC proteins in the cultures supernatant. Western blot analysis of GtfB and GtfC enzymes indicated that significantly less Gtfs were present in the cultures of *S. mutans* grown in the presence of HDA than for cells grown without treatment (Figure 4). Ooshima and colleagues reported that an optimal GtfB/GtfC ratio is necessary for appropriate colonization *in vitro*[41], hence divergence from this proportional relation could compromise the adherence of the treated bacteria to the cells as seen in Table 1.

Previously we found that high concentrations of RJ could inhibit the growth of *S. mutans* but also in lower concentrations it was inhibited *gtf* genes expression [42]. Findings of this study implied that HDA could penetrate into *S. mutans* and kill the organism as tested by time-kill kinetic assay (Figure 2). The mechanism by which the HDA inhibits the expression of *gtfB* and *gtfC* and decreases water insoluble glucan is probably related to the binding to their promoters, blocking RNA synthesis and their expression.

The down regulation of the *gtfB* and *gtfC* genes is responsible for the easily detachable biofilm phenotype and decrease in the attachment power of the organisms to the P19 cell surfaces (Figure 5C) in comparison to the untreated bacterial cultures (Figure 5B). By reduction in glucan levels there is no more binding substrate that may prohibits α-1,6 glucan dependent biomass aggregation. It has been shown that HDA stimulates collagen production and enhances deposition of collagen in the dermis [43]. Our investigation revealed that HDA decreased the proliferation of P19 embryonic cells but morphologic changes also occurred while neuron like cells were observed by phase contrast microscopy (Figure 5A). After HDA treatment of *S. mutans* the invasion of P19 embryonal cells was decreased after 6 h of incubation under the growth conditions described previously (Table 1). Concentration of 500 μg ml-1 of HDA could inhibit adherence of *S. mutans* by 59% (Table 1). By preventing adhesion of pathogenic bacteria to their host cells; decreases amount of colonization and in this way reduces the extent of pathogenicity.

The P19 embryonic cell invasion assay developed for testing of HDA towards selected *S. mutans* represents an attempt to recreate environmental conditions that could be compared to the other eukaryotic cells, and may develop a new model for testing the effect of pharmaceutical compounds on pathogens.

## Conclusion

In conclusion 10-hydroxy-2-decenoic acid treatment could down-regulate the *gtfB* and *gtfC* genes expression in *S. mutans.* Also it could decrease adherence of *S. mutans* to the P19 cells surfaces. Future studies will focus on differential display PCR and microarray analysis to reveal additional *S. mutans* genes that are subjected to HDA effects. This hypothesis will encourage our understanding of gene regulation and signal transduction in *S. mutans*, and facilitate the development of therapeutic approaches to control formation of the plaque biofilms and dental caries.

## Abbreviations

HDA: Hydroxy decenoic acid; RJ: Royal jelly; *S. mutans*:* Streptococcus mutans*; Gtf: Glucosyltransferase; RT-PCR: Reverse transcriptase polymerase chain reaction; HRP: Horse reddish peroxidase; DMEM: Dulbecco’s Modified Eagle’s Medium; FCS: Fetal Calf Serum; PBS: Phosphate Buffer saline.

## Competing interests

The authors declare that they have no competing interests.

## Authors’ contributions

BY performed culture, treatment and gene extraction. SG designed experiments, interpreted results. ARL performed SDS PAGE and Western blot analysis. NA designed primers, performed real time PCR and interpreted gene expression profile. JV interpreted results, drafted manuscript. ASH conducted experiments, Critical revision to manuscript, and final draft of manuscript. All authors read and approved the final manuscript.
